# Integrated bio-cooperative robotic platform for virtual cognitive training in Parkinson's disease: design and methodology of the OPERA project

**DOI:** 10.3389/fneur.2025.1680215

**Published:** 2026-01-26

**Authors:** Cristina Polito, Giulia Martinelli, Sara Della Bella, Eleonora Pavan, Ylenia Crocetto, Simona Abagnale, Cristiana Rondoni, Alfonso Voscarelli, Marco Pirini, Francesco Scotto di Luzio, Loredana Zollo, Anna Estraneo

**Affiliations:** 1 Istituto di Ricovero e Cura a Carattere Scientifico (IRCCS) Fondazione Don Carlo Gnocchi, Florence, Italy; 2 Research Unit of Advanced Robotics and Human-Centred Technologies, Università Campus Bio-Medico di Roma, Rome, Italy; 3 Khymeia Group, Padua, Italy

**Keywords:** Parkinson's disease, mild cognitive impairment—MCI, cognitive training, virtual reality, robot-aided rehabilitation, multimodal platforms, bio-cooperative systems

## Abstract

**Introduction:**

Mild cognitive impairment in Parkinson's disease (PD-MCI) can affect several cognitive domains, including attention, working memory, executive functions, language, visuospatial skills, and episodic memory, resulting in a progressive reduction of autonomy and an increased risk of dementia. Cognitive training may help preserve cognitive abilities, especially when supported by innovative tools; nevertheless, standardized and engaging interventions are still lacking. The OPERA project aims to develop and evaluate the usability of PRoBio, a novel bio-cooperative platform that integrates virtual reality (VR), robotic assistance and physiological monitoring to deliver personalized cognitive rehabilitation for individuals with PD-MCI.

**Methods and analysis:**

The OPERA project is a 13-months non-profit, multicentre clinical investigation structured in four phases. Phase 1 (month 2): focus group, involving 23 participants (10 people with PD (PwPD), 5 caregivers, 8 healthcare professionals) to explore usability, expectations and rehabilitation needs. Phase 2 (months 2–7): development of the PRoBio platform, by integrating the “Virtual Reality Rehabilitation System” (VRRS, by Khymeia Group) with the TIAGo robot (by PAL Robotics) to deliver personalized exercises to patients' cognitive profiles, while also monitoring their emotional and physiological state. Phase 3 (month 6): two living labs involving a total of 21 healthy subjects (13 volunteers and 8 rehabilitation professionals) to assess PRoBio's usability in a real setting, with emotional data collection and standardized usability questionnaires completion after use. Phase 4 (months 8–12): usability study assessing PRoBio's usability as the primary objective, involving 10 PD-MCI patients completing a 4-week cognitive rehabilitation program with pre/post clinical and neuropsychological assessments. Descriptive statistics and appropriate inferential tests (parametric or non-parametric) will be applied to usability data, pre/post intervention clinical measures, and physiological and performance data registered by the PRoBio platform (*p* < 0.05).

**Conclusion:**

The present paper presents the methodological framework of the OPERA project, which brings together partners with complementary expertise to develop and evaluate the PRoBio platform, a novel bio-cooperative system for cognitive rehabilitation in patients with PD-MCI. By integrating VR, robotics and physiological feedback, PRoBio aims to enable personalized, adaptive interventions, offering a more engaging alternative to traditional rehabilitation approaches while advancing research in bidirectional human–robot interaction.

## 1 Introduction

Parkinson's disease (PD) is a progressive neurodegenerative disorder, characterized by cardinal motor symptoms (rigidity, resting tremor, and bradykinesia) that in the early stages predominantly affect one side of the body [Movement Disorder Society (MDS)] (1). In addition to its motor features, PD is associated with a wide spectrum of non-motor symptoms. Among these, Mild Cognitive Impairment (MCI) is a frequent and clinically relevant condition that can involve several cognitive domains such as attention, executive functions, working memory, and visuo-spatial abilities (2, 3). Up to 60% of MCI in Parkinson's Disease (PD-MCI) may progress to Parkinson's Disease Dementia (PDD) (4) within 5 years from onset (3). A recent meta-analysis on studies using MDS criteria estimated a pooled prevalence of PD-MCI at ~40% (5). Further, longitudinal studies suggested that 20%−30% of patients are diagnosed with MCI at the time of their first clinical diagnosis of PD, 40%−50% within 5 years, and up to 75% after more than 10 years of disease (3, 6).

The presence of cognitive impairment in PD has been shown to significantly impact a patient's functional autonomy and daily functioning and hamper the effectiveness of motor rehabilitation (7). Despite the high prevalence and clinical relevance of PD-MCI, there is a paucity of standardized and evidence-based cognitive rehabilitation approaches and protocols tailored to this population.

Although the limited number of available studies and the differences in methodology, a Cochrane review reported preliminary evidence supporting the feasibility and potential benefits of cognitive training on global cognition in individuals with PD-MCI or PDD (8). The review further highlighted the lack of studies on long-term and functional outcomes, underscoring the need for large-scale, rigorous trials assessing both cognitive and functional outcomes. A more recent systematic review on cognitive training in people with PD (PwPD) and with PD-MCI selected 28 randomized controlled trials or clinical trials (9). The interventions included structured paper-and-pencil training, computer-based platforms for exergames, virtual reality (VR), dual-task paradigms, action observation therapy, and motor imagery techniques. Most studies reported a modest and inconsistent overall cognitive improvement after training; some found a significant improvement in specific cognitive domains, particularly in attention and memory, whereas effects on executive functions were more inconsistent. These findings could be ascribed to methodological issues, such as the small samples employed, the absence of long-term follow-up and of a control group of participants who did not undergo training, or the presence of additional elements in some trials, like psychoeducation or unspecific general rehabilitation aspects.

Among rehabilitative interventions, VR-based training has been proposed to enhance patient engagement through ecologically valid training scenarios. Via multimodal stimulation, VR can closely mimic real-life activities not easily reproduced in traditional rehabilitation settings. In addition, real-time visual and auditory feedback provided by VR systems may support increased motivation and adherence to treatment. VR-based cognitive rehabilitation is a rapidly evolving field in PD, with several European clinical studies and research projects demonstrating its feasibility and efficacy. Recent studies have demonstrated the efficacy of VR interventions, encompassing immersive and semi-immersive platforms such as the Computer Assisted Rehabilitation Environment (CAREN) and the BTS Nirvana, in enhancing executive function, memory, visuospatial abilities, and emotional domains in PwPD, particularly those exhibiting MCI (10–14). Typically, VR-based cognitive rehabilitation is delivered through structured sessions conducted either in clinical settings or remotely via tele-medicine. The integration of virtual coach technology within tele-rehabilitation platforms provides interactive, adaptive feedback and personalized guidance to patients (14–16). Home-based digital cognitive interventions improve adherence and accessibility, reducing barriers related to distance, time, and cost, with overall costs comparable to or lower than traditional in-person rehabilitation (16–18). Despite these promising outcomes, current VR-based studies are still limited by small sample sizes, heterogeneous intervention protocols, and short follow-up durations (9).

Concurrent with the implementation of VR in clinical and tele-rehabilitation settings, innovative tools and technologies leveraging machine learning and robotics have been successfully introduced in the field of psychology (19, 20). Robotics has found significant applications, particularly in rehabilitation and care. Specifically, social robots are increasingly used in psychological, cognitive-motor rehabilitation, where emotional and social cues are key to effective interactions. Objective insights into emotional engagement emerged from automated analysis of spontaneous facial expressions during human–robot interactions (21).

Recent advances in rehabilitation are moving toward the development of adaptive and bio-cooperative rehabilitation systems that integrate physiological and behavioral measurements to personalize therapy in real time. These systems use a “*human-in-the-loop”* model, whereby the user's psychophysiological state directly informs system adaptation, promoting more effective, user-centered interventions. Multimodal adaptive interfaces combining motion tracking, heart rate (HR), and galvanic skin response (GSR), have been proposed to guide robotic assistance (22, 23). These robotic-mediated rehabilitation interfaces demonstrate that integrating biosignals enables dynamic adjustments of task difficulty and robotic support. Similarly, Cisnal et al. (24) developed an embedded bio-cooperative platform that integrates electrocardiography (ECG), GSR, electromyography (EMG), and inertial sensors for real-time adaptation in neuromotor rehabilitation, confirming the feasibility of bio-cooperative control. Recent works have further proposed bio-cooperative robotic platforms to deliver tailored rehabilitation through a bidirectional exchange of information with the user (6, 25).

Despite these advances, most bio-cooperative systems focus on motor rehabilitation, where adaptation affects mechanical assistance or movement dynamics. Few studies, however, have explored bio-cooperative principles in cognitive or affective rehabilitation, where adaptation could modulate task complexity, cognitive load, or motivational feedback. As described above, VR has emerged as a powerful tool for neurorehabilitation, providing engaging and ecologically valid environments that can enhance neuroplasticity and cognitive recovery (26). However, VR-based systems often remain open-loop, offering limited responsiveness to the psychophysiological state of the user. Recent works have also underscored the importance of psychophysiological markers as reliable indicators of engagement, stress, and workload during human–robot interaction. Catalán et al. (27), for example, showed that competitive robotic tasks elicit significant changes in HR and GSR, suggesting that biosignals can be used as meaningful proxies of cognitive effort and emotional involvement in rehabilitation contexts. Such findings support the potential of integrating real-time physiological monitoring into adaptive therapeutic systems that can modulate not only physical assistance but also cognitive and emotional engagement.

To address this gap, the present paper describes the methodological framework of the OPERA project (“Integrated biO-cooPErative Robotic plAtform for virtual cognitive training in Parkinson's Disease”). The main objectives of the project are to design, develop, and test the usability of PRoBio, a novel bio-cooperative rehabilitation platform that integrates VR-based cognitive training with robotic assistance, able to tailor the treatment to the psychophysiological state of the user. This human-in-loop approach seeks to transform the rehabilitation platform into an active, closed-loop process that is also responsive to fluctuations in engagement and emotional state.

## 2 Methods and analysis

**Figure 1:**
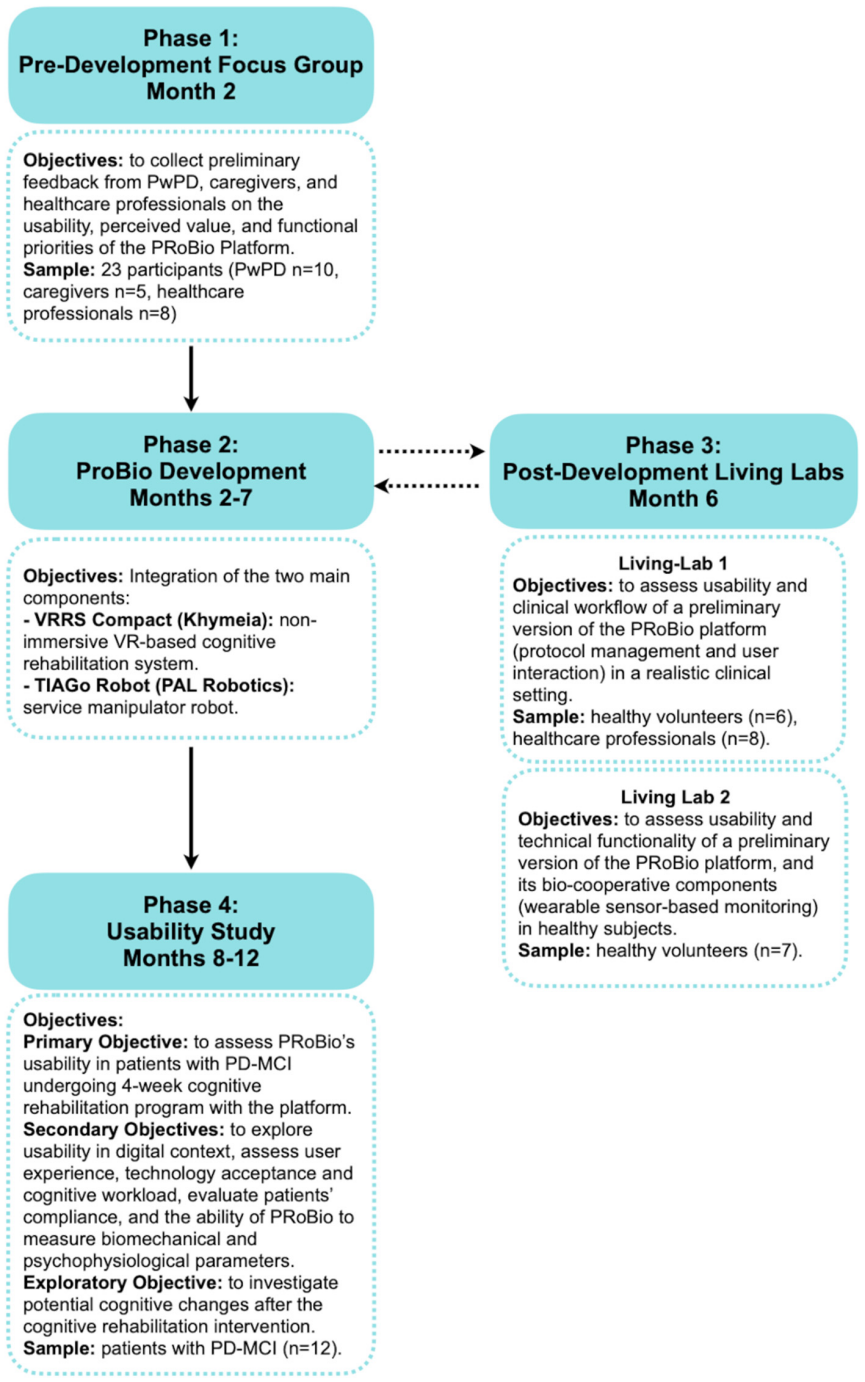
Description of the four main phases of the OPERA project, with their objectives and specific characteristics, and expected timeline. PwPD, People with Parkinson's Disease; PRoBio, Bio-cooperative Robotic Platform; VRRS, Virtual Reality Rehabilitation System; PD-MCI, Parkinson's Disease with Mild Cognitive Impairment.

The OPERA project brings together three partners with complementary expertise: (1) Don Carlo Gnocchi Foundation (Sant'Angelo dei Lombardi and Florence sites, Italy) as clinical partners, (2) The Research Unit of Advanced Robotics and Human-Centered Technologies of Università Campus Bio-Medico di Roma (UCBM, CREO Lab, Italy) and (3) Khymeia SRL (Italy) as technological partners. The project has been conducted over 13 months (October 2024-October 2025) through four main phases (Figure 1):

Phase 1 (Month 2)—Pre-PRoBio development Focus group: structured session with patients, caregivers, and healthcare professionals to define user needs and functional requirements for the PRoBio platform.

Phase 2 (Months 2–7)—PRoBio development: technical integration of robotic, virtual, and physiological modules into a unified platform.

Phase 3 (Month 6)—Post-PRoBio development Living Labs: usability testing with healthy participants to assess system performance and refine interaction modalities of PRoBio platform.

Phase 4 (Months 8–12)—Usability study: evaluation of the platform's usability by patients with PD-MCI undergoing 4-weeks cognitive training with PRoBio in clinical setting.

At the time of the initial submission of this methodological paper, Phases 1–3 of the OPERA project had been completed, and technical refinements of the PRoBio platform were being implemented to enable the initiation of Phase 4 (usability testing in PD-MCI participants). At the time of publication, the OPERA project has been concluded, as the “Age-It–Aging well in an aging society” project (PE0000015), CUP B83C22004800006, National Recovery and Resilience Plan (NRRP)–PE8–Mission 4, C2, Intervention 1.3 officially ended on October 31, 2025. The present article was conceptualized with the intention of providing a detailed description of the design and methodology prior to the completion of the study, nonetheless, due to project time constraints, the final revision of this publication was finalized after the project conclusion.

### 2.1 Phase 1. Pre-PRoBio development Focus Group

#### 2.1.1 Study design

Phase 1 consisted of a single session Focus Group to evaluate usability, acceptability, and perceived value of the new technology (PRoBio), facilitating iterative refinement prior to large-scale deployment, according to user-center design.

#### 2.1.2 Methods

*Objectives*. The objective of the Focus Group was to collect preliminary feedback from PD patients, caregivers, and healthcare professionals on the usability, perceived value, and functional priorities of the PRoBio platform prior to its development. This user-centered step aimed to inform the design process and ensure the platform addresses real-world rehabilitation needs.

*Study participants*. Twenty-three participants have been recruited and distributed across three groups: (1) PwPD (*n* = 10; *F* = 6; mean age = 68.9 years ± 8.2); (2) informal or family caregivers of PwPD (*n* = 5; *F* = 3; mean age = 49.0 years ± 15.5); (3) healthcare professionals involved in PD management (*n* = 8; *F* = 6; mean age: 40.0 years ± 12.0), representing the direct end-users of the system.

*Procedures*. Participants attended a structured session introducing the OPERA project and the PRoBio platform, including explanatory videos on the robotic and VR components. At the end of the session, all participants completed a structured, group-specific *ad-hoc* questionnaire to assess user perceptions of the project's goals, perceived usability, usefulness, relevance, and impact of the technological devices (VR and robot) and their potential combination into a unified platform for rehabilitation.

For each participant, sociodemographic information has been collected. In addition, disease duration (for patients), caregiving experience (for caregivers), and professional role and years of clinical experience with PD (for healthcare professionals) have been recorded.

*Sample size*. No *a priori* sample size calculation was performed, as Focus Groups aim to collect qualitative insights rather than statistical representativeness (28).

#### 2.1.3 Statistical analysis

Descriptive statistics were computed (means, standard deviations, and frequencies) on: (a) demographic and clinical data of the participants for each group (i.e., age, sex, disease duration, caregiving experience, and professional role and years of clinical experience with PD); (b) *ad-hoc* questionnaires. Chi-square was used to assess differences in perceptions among the three groups (PwPD, caregivers, and healthcare professionals). The level of significance was set at *p* < 0.05 for all analyses, which were carried out with Jamovi (version 2.5, 2024) (29).

For details regarding statistical analysis and results of Phase 1—Focus Group see Crocetto et al.'s (30) study.

### 2.2 Phase 2. PRoBio development

#### 2.2.1 PRoBio Platform and its main features

**Figure 2:**
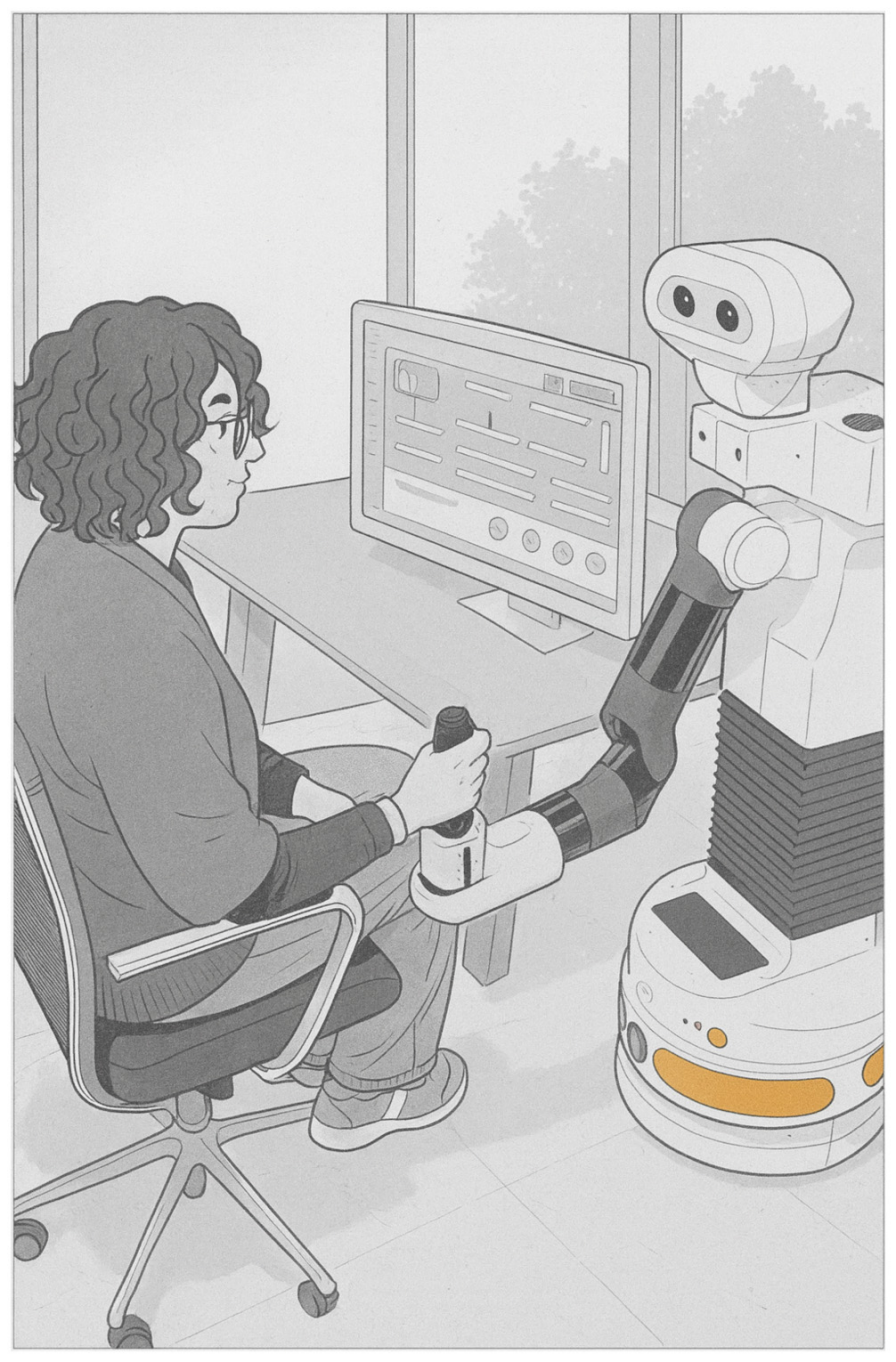
Schematic representation of the PRoBio platform, integrating VR-based cognitive training, robotic assistance (TIAGo), and physiological signal monitoring for personalized rehabilitation in PD-MCI. Illustration generated with ChatGPT-4o based on an original photograph taken by the authors. The original photograph can be provided by the authors upon request.

The PRoBio platform was developed in accordance with the insights derived from the Focus Group. It is a bio-cooperative platform (Figure 2), designed to deliver personalized cognitive rehabilitation by leveraging multimodal feedback to adapt therapeutic interventions to the cognitive and psychophysiological profile of each patient. It is composed of the following core modules:

1. TIAGo robot (PAL Robotics SL, Spain), a mobile robotic platform equipped with a seven-DOF anthropomorphic arm, an RGB-D camera embedded in a pan-tilt head, an adjustable-height torso, and a mobile base. The robot also includes microphones and speakers for vocal interaction, and an end-effector with a two-finger gripper (robotic arm). TIAGo acts as the main interaction unit between the participant and the rehabilitation system, operating in two modes: (i) Joystick mode, in which forces applied by the user to the closed gripper are translated into compliant Cartesian displacements for motor activation and kinematic analysis; (ii) Monitoring mode, where the RGB-D camera enables skeletal tracking and facial expression analysis, allowing the detection of seven categories of emotions. The robot also employs a Text-to-Speech engine to deliver instructions and real-time feedback to the participant. Its behavior is coordinated by a Finite State Machine (FSM), which governs transitions among operational states;
2. VRRS Compact (Khymeia SRL, Italy), a VR module, which includes a central processing unit and a capacitive touchscreen LCD display. It enables non-immersive virtual rehabilitation scenarios, offering a safe, customizable environment for motor and cognitive exercises; and
3. PRoBio Multimodal Architecture, for biomechanical and psychophysiological monitoring. It is designed to acquire, process, and integrate heterogeneous physiological and biomechanical signals in real time and implemented within the Robot Operating System (ROS) framework, enabling modularity, scalability, and interoperability among components. The architecture incorporates data from the RGB-D camera of the TIAGo robot and two wearable sensors (Zephyr Bioharness for monitoring heart and respiratory rate and Shimmer GSR+ sensor for recording galvanic skin response, a proxy for sympathetic nervous system activity). This architecture processes multimodal data in real time to estimate posture, arousal, valence, and facial expressions. It also analyses vocal cues captured by the microphones of the robot, which can indicate effort, frustration, or engagement. Moreover, the multimodal platform is also equipped with an adaptive algorithm, which modulates the interaction between the user and the system based on continuous monitoring of physiological and behavioral parameters. At the start of each session, a brief calibration phase is performed to establish a subject-specific physiological baseline. During this phase, HR, RR, and GSR are recorded under resting conditions to compute mean and standard deviation values. These baseline parameters are then used to normalize subsequent physiological data, allowing the system to detect relative changes in the state rather than relying on fixed thresholds. During the execution of cognitive exercises, physiological features are continuously extracted from HR, RR and GSR using rolling 1-s windows and combined with facial-expression features obtained from the RGB-D camera, which captures basic emotional states. The system also analyses vocal cues recorded by the robot, which can signal effort, frustration, or engagement. These multimodal inputs are implemented within the ROS environment and processed in real time. All the acquired data are processed in real time and displayed through a dedicated graphical interface, which allows the clinician to observe the evolution of the quantitative indicators during the rehabilitation session. This continuous visualization enables constant supervision of the behavior of the platform and of the interaction with the patient, supporting timely adjustments of therapeutic strategies and ensuring safe and effective operation of the system.

#### 2.2.2 Cognitive training by PRoBio

Two 45-min PRoBio-based cognitive training protocols (A and B) have been created by selecting cognitive exercises already available on the Khymeia VRRS system. Each protocol consists of 14 exercises, with an equal number of tasks targeting the same cognitive domains (i.e., attention, executive functions, memory, and visuospatial skills) as shown in Table 1. The protocols were designed by selecting exercises according to the type of task and the targeted cognitive component. For each cognitive function, comparable exercises were distributed across the two protocols to maintain balance in terms of duration, sequencing, and input modality (touchscreen or robotic arm). A more detailed description of the protocols and exercises is shown in Supplementary Table 1.

**Table 1:** Brief description of the two rehabilitation protocols (A and B).

**Cognitive domain**	**Protocol A (Khymeia's task name)**	**Protocol B (Khymeia's task name)**	**Modality**
Attention	Find the differences	Attentive matrix	Touch screen
	Car	UFO	Robotic arm
	Umbrella	Connect the point (TMT)	Robotic arm (A)/Touch screen (B)
	Changing color	No changes	Touch screen
	Changing shape	Changing everything	Touch screen
	Reaction times	Reaction times	Touch screen
Executive function	Sequence of shapes	Get money up to 10€/100€/1,000€/1€	Touch screen
	Plannings	Sort the images in series	Touch screen
	Street path	Daily sequences	Touch screen
	Complete the series	Dual category	Touch screen
	Stroop test	Alphanumeric sequencing	Touch screen (A)/Robotic arm (B)
Visuospatial	Find the symmetric item	Recognize the object among many	Touch screen
	Guess the rotation direction	Puzzle	Touch screen
Memory	Words memory	Long term memory	Touch screen

List of the selected specific tasks for each domain and, brief description of the interaction modality used—either touch screen or robotic arm. More detailed information and descriptions can be found in Supplementary Table 1.

TMT, trail making test; UFO, unidentified flying object.

### 2.3 Phase 3. Post-PRoBio development Living Labs

#### 2.3.1 Study design

Phase 3 of the OPERA project consisted of two single-session Living Labs. The first was conducted in a clinical setting at the Don Carlo Gnocchi Foundation with healthy volunteers and rehabilitation professionals, while the second took place in a research laboratory at Università Campus Bio-Medico di Roma (Italy), where the platform was developed, involving healthy participants according to Living Labs methodology (31–33).

#### 2.3.2 Methods

*Objectives*. The primary objective of the Living Labs was to evaluate a preliminary version of the PRoBio platform, specifically the integration of the VRRS module and the TIAGo robot, in real world settings. The focus of the first living lab was on usability and clinical workflow (protocol management and user interaction), while the bio-cooperative components (e.g., wearable sensor-based monitoring) have been assessed separately in the second Living Lab with healthy volunteers. The findings from the two Living Lab sessions informed and guided the re-designed version of PRoBio.

*Study participants*. Inclusion criteria for the participants were age of at least 18 years and no history of neurological, cognitive, or psychiatric disorders. All participants provided informed consent to participate in the study. In the Living Lab 1 we enrolled six healthy volunteers (*F* = 3; mean age = 50.3 ± 11.0 years) and eight rehabilitation professionals (*F* = 3; mean age = 32.8 ± 9.9 years). In the Living Lab 2 we enrolled seven healthy volunteers (*F* = 4; mean age = 27.2 ± 4.4 years). The Living Lab phase intentionally excluded patients with PD-MCI and involved only healthy volunteers and healthcare professionals. This methodological choice was made to frame the study as a pre-clinical validation step, aimed at verifying the technical reliability, usability, and safety of the system before vulnerable patient exposure.

*Procedures*. Healthy subjects participated in a single interaction session with the PRoBio platform. Each participant was asked to perform two cognitive tasks delivered through the VRRS: one using the touchscreen mode (1st task) and one using the robotic arm as a joystick (2nd task). The VRRS provided auditory feedback on task performance, and recorded parameters of the user's performance (e.g., reaction time), while the robot monitored facial expressions through an RGB-D camera and delivered vocal prompts to improve user engagement.

Immediately after their interaction with the system, in order to minimize memory bias effects, participants completed questionnaires to explore five main domains: (i) general usability, (ii) user experience, (iii) workload, (iv) acceptance and intention to use, and (v) eHealth-specific aspects. Accordingly, the following validated instruments were administered: (i) System Usability Scale (SUS) (34) as a standardized index of usability; (ii) eHealth UsaBility Benchmarking Instrument (HUBBI) (35) to capture aspects specific to digital health services such as trust, safety, and information quality; (iii) Short Version of the User Experience Questionnaire (UEQ-S) (36) to assess pragmatic qualities of the interaction; (iv) Scales for Perceived Usefulness and Perceived Ease of Use (TAM) (37) to evaluate perceived usefulness and ease of use as predictors of behavioral intention; (v) the Italian version of the Unified Theory of Acceptance and Use of Technology (I-UTAUT—Original version proposed by Venkatesh (38), later adapted for the field of assistive robotics by Heerink (39) and finally translated and validated in Italian by D'Iorio (40)) to measure performance expectancy, social influence, and facilitating conditions; (vi) NASA Task Load Index (NASA-TLX) (41) to quantify multidimensional workload as a potential limiting factor in cognitive rehabilitation. See the description of the questionnaires in the Supplementary materials.

Italian versions were used whenever available, and short-form versions were preferred to minimize respondent burden. Total completion time was 10–15 min. The entire Living Lab session lasted ~1 h, including the initial interaction with the PRoBio system and the subsequent completion of the usability questionnaires by the participants.

Information on age, sex, educational level, handedness preference and professional role (for healthcare professionals) has been collected.

*Sample size*. Based on the methodology of the Living Lab, the estimation of a specific sample size is not mandatory as its primary objective is to explore and optimize the interaction of the user with the platform in a real scenario (42).

#### 2.3.3 Statistical analysis

Descriptive statistics (means, standard deviations, and frequencies) were computed on (a) demographic and clinical data of the participants (i.e., age, sex, healthcare profession); (b) standardized usability questionnaires. Exploratory group comparisons were performed. All statistical analyses were performed using Jamovi software (version 2.5, 2024) (29).

Further details on the statistical analysis and results from Phase 3—Living Lab 1 can be found in Crocetto et al. (30).

### 2.4 Phase 4. Usability study

Phase 4 consisted of a multicentre, non-CE-marked medical device clinical investigation aimed at assessing the usability of the PRoBio integrated platform, refined based on feedback from the Living Labs.

#### 2.4.1 Methods

*Objectives*. Objectives of Phase 4 were:

- Primary objective: to evaluate the usability of PRoBio in a group of patients with PD-MCI undergoing a structured 4-week cognitive rehabilitation program delivered by the platform.
- Secondary objectives: (1) to further explore usability in a digital health context, and to assess the user experience, technology acceptance and cognitive workload during the interaction with PRoBio; (2) to assess the acceptability and compliance with the treatment among patients with PD-MCI; (3) to assess the acceptability of the PRoBio platform among rehabilitation professionals; (4) to evaluate PRoBio's capability to record and analyse psychophysiological parameters via RGB-D camera and wearable sensors; (5) to evaluate PRoBio's capability to collect real-time performance data (accuracy and response times) during cognitive exercises administered via VRRS.
- Exploratory objective: to investigate the potential cognitive changes after a 4-week cognitive rehabilitation intervention delivered via PRoBio in patients with PD-MCI.

*Study participants*. PwPD have been recruited from outpatients attending the Don Carlo Gnocchi Foundation Rehabilitation Center in Sant'Angelo dei Lombardi and Florence sites, or upon referrals from physicians at other centers previously informed about the study. Patients had to meet the following inclusion criteria: (i) a minimum age of 18 years; (ii) diagnosis of PD according to MDS criteria (1); (iii) Hoehn and Yahr stage I–II (43); (iv) MCI according to Litvan criteria (2); (v) provision of written informed consent to participate in the study.

Individuals were excluded if they had: (i) severe depression; (ii) PDD; (iii) atypical Parkinsonism, secondary mixed forms; and (iv) severe behavioral and cognitive disorders associated with other diseases, including delirium and stroke.

*Procedures*. Enrolled patients underwent a comprehensive clinical and neuropsychological assessment to evaluate motor, cognitive, and behavioral functioning at baseline (T0). Following the assessment, all participants were engaged in a 45-min PRoBio-based cognitive training three times per week for four consecutive weeks. The program followed an ABAB design, in which two balanced protocols (A and B, as detailed in Section 2.2.2) alternated on a weekly basis. Every week for each protocol, the complexity of the exercises was modified based on the patient's performance during the last session of the previous week to ensure a tailored difficulty improvement on a weekly basis. At the end of the 4-weeks treatment (T1) patients completed the usability and acceptability questionnaires and underwent the same clinical and neuropsychological assessment performed at baseline.

Age, sex, educational level, handedness, disease stage according to Hoehn and Yahr criteria (43), disease duration, and the predominantly affected hemi-body were collected for each participant.

*Assessment tools*. Clinical and neuropsychological assessment performed at T0 and T1 included the following standardized tools (for details see Supplementary materials):

- MDS-Unified Parkinson's Disease Rating Scale (MDS-UPDRS) (44).
- Parkinson's Disease Cognitive Rating Scale (PD-CRS) (45).
- Stroop Color Word Test (46).
- Trail Making Test (TMT) (47).
- Parkinson's Disease Questionnaire (PDQ-39) (48).
- 15-item Geriatric Depression Scale (GDS-15) (49, 50).
- Geriatric Anxiety Inventory (GAI) (51).

**Figure 3:**
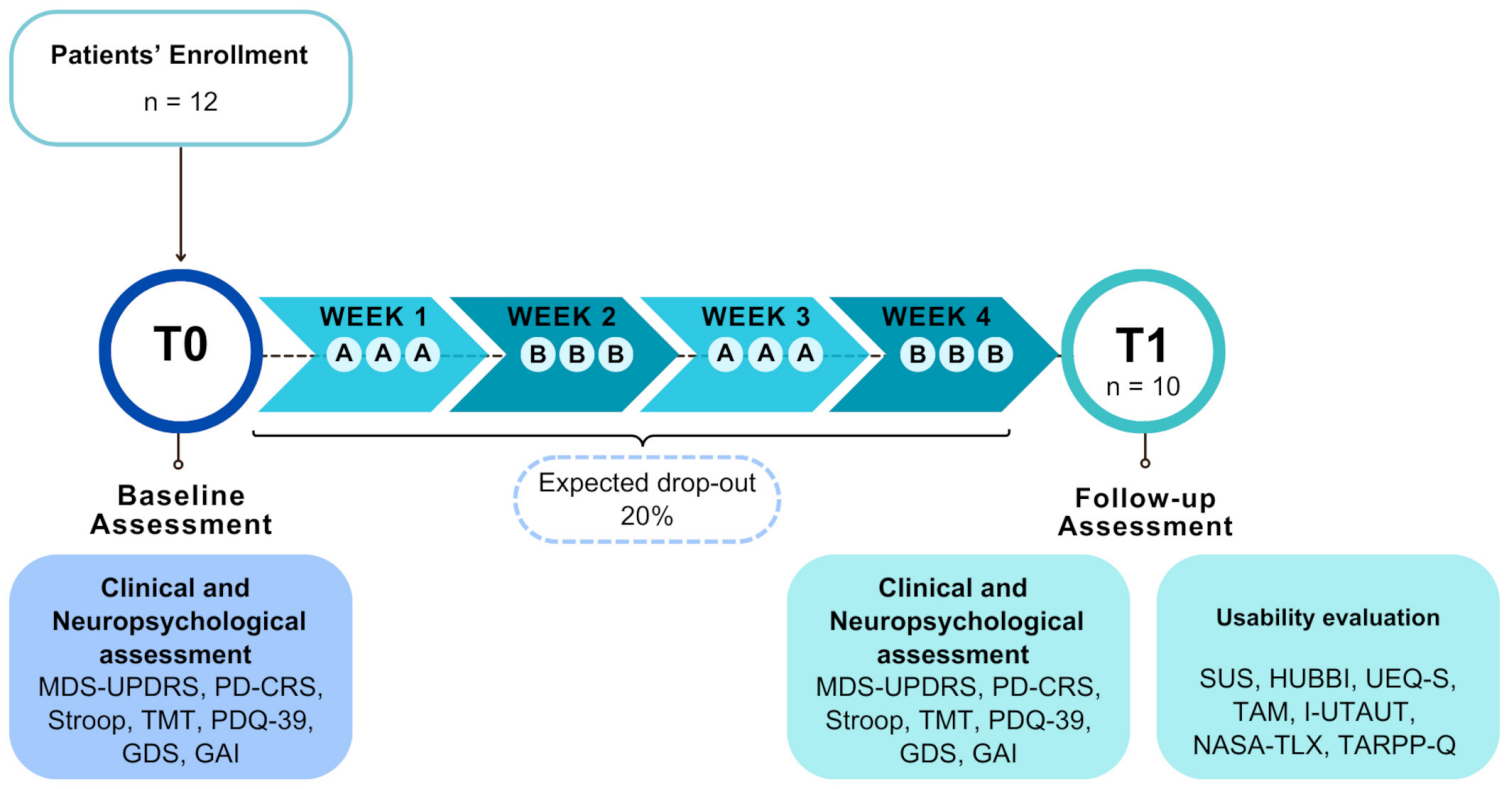
Graphic representation of the Usability Study procedures and the data flow within the OPERA project. Clinical and neuropsychological data are collected at baseline (T0), and at the end of the treatment (T1). Usability data are collected at T1. T0, Baseline Assessment; T1, Follow-up Assessment; MDS-UPDRS, Movement Disorder Society—Unified Parkinson's Disease Rating Scale; PD-CRS, Parkinson's Disease-Cognitive Rating Scale; TMT, Trail Making Test; PDQ-39, Parkinson's Disease Questionnaire; GDS, Geriatric Depression Scale; GAI, Geriatric Anxiety Inventory; SUS, System Usability Scale; HUBBI, eHealth UsaBility Benchmarking Instrument; UEQ-S, User Experience Questionnaire-Short version; TAM, Technology Acceptance Model; I-UTAUT, Unified Theory of Acceptance and Use of Technology-Italian version; NASA-TLX, NASA Task Load Index; TARPP-Q, Technology Assisted Rehabilitation Patient Perception Questionnaire.

At T1, patients completed the set of usability and acceptability questionnaires used in the Living Lab phase (see Section 2.3.2), which have been adapted for patients. An additional Technology Assisted Rehabilitation Patient Perception questionnaire (TARPP-q) (52) was administered to evaluate the patients' perception of technology-assisted rehabilitation. The graphic representation of the usability study procedures is shown in Figure 3.

*Outcome measures*.

Primary outcome: usability of PRoBio assessed by SUS (34).

Secondary outcomes:

- Specific aspects of usability of digital health services such as trust, safety, and information quality, measured with the HUBBI (35). User experience, including pragmatic and hedonic quality, measured with the UEQ-S (36) and with the TARPP-q (52). Technology acceptance explored by the TAM (37) and the I-UTAUT (40). Cognitive workload during interaction assessed with the NASA-TLX (41).
- Acceptability of the PRoBio platform and compliance with the treatment among patients with PD-MCI measured by (a) the number of treatment sessions completed/number of treatment sessions planned, (b) the number of treatment weeks/total number of weeks planned.
- Acceptability of the PRoBio platform among rehabilitation professionals measured by (a) the mean time for wearable sensors positioning and starting of the system; (b) the number of system errors/warning; (c) the percentage of sessions' completion.
- Biomechanical parameters (e.g., range and speed of the patient's movement) recorded by the RGB-D camera, and psychophysiological parameters (e.g., respiratory rate—RR, HR, and GSC), measured through wearable sensors during the treatment sessions.
- PRoBio's capability to collect real-time performance data during cognitive exercises administered via VRRS assessed by the number of output files recorded by PRoBio/number of exercises per session.

Exploratory outcome: changes in clinical (MDS-UPDRS) and neuropsychological variables (PD-CRS, Stroop Test and Trail Making Test, GDS, GAI), and quality of life (PDQ-39) between the two timepoints T0 and T1.

*Sample size*. The sample size was derived using data from Saric et al. (53), who reported a mean SUS score of 77.5 (IQR: 70–80.625) when evaluating the usability of a sensor-based interactive hand device. Assuming a 95% confidence level and a conservative margin of error of five points, the minimum required sample was 10 participants, increased to 12 to mitigate potential data loss.

Since the primary objective was to assess the system's usability, all participants underwent treatment with the platform, and no control group was enrolled.

#### 2.4.2 Statistical analysis

All statistical analyses will be conducted using Jamovi software (version 2.5, 2024) (29). The Shapiro-Wilk test will be conducted to explore the distribution of the data. Descriptive statistics will be performed on primary and secondary outcomes' measures. Continuous variables will be presented as mean (standard deviation—SD) or median (interquartile range—IQR), as appropriate, and for categorical variables, as frequencies or percentages.

Paired *t*-tests, or Wilcoxon signed-rank test, as appropriate, will be performed to assess differences in clinical, neuropsychological and quality of life measures before and after the intervention (exploratory outcome).

The significance level for all analyses will be set at *p*-value < 0.05.

The data acquired from the PRoBio platform will be processed through a structured workflow. In the first stage, raw data from all sensors will be pre-processed to remove artifacts. Outlier detection will be performed using robust statistical methods to ensure data integrity. In the second stage, a comprehensive set of features will be extracted. For cardiorespiratory activity, time-domain metrics will include HR and RR. For electrodermal activity, features derived from GSR will include tonic (Skin Conductance Level, SCL) and phasic components (Skin Conductance Responses, SCR), as well as frequency and amplitude of SCRs over time windows. Data acquired from the RGB-D camera and robot sensors will be used to compute biomechanical metrics. For facial expression analysis, discrete emotional states (e.g., neutral, happy, sad, surprised) will be inferred using facial action coding systems and mapped to dimensional descriptors such as valence and arousal. For all extracted features, descriptive statistics will be computed across sessions and participants. These metrics will be used both for individual-level profiling and for group-level comparisons in subsequent inferential analyses. The data will be segmented into three predefined time windows within each session, namely baseline, active interaction, and recovery, to facilitate intra-session comparison. Subsequent statistical analyses will be produced by the data distribution and will include both parametric and non-parametric tests. Furthermore, to investigate the interaction and combined effects of multiple variables (e.g., interaction duration and the participant's emotional state), linear mixed models (LMMs) or repeated measures analysis of variance (RMANOVA) will be employed. These models will facilitate the incorporation of within-subject and between-subjects factors. Statistical significance will be set at *p* < 0.05. Where relevant, corrections for multiple comparisons will be implemented using the Bonferroni method and the False Discovery Rate (FDR) approach.

## 3 Discussion

The present methodological paper outlines the step-by-step procedures adopted into the development and preliminary testing of a novel, bio-cooperative rehabilitation platform (PRoBio), designed to deliver adaptive cognitive training tailored to the cognitive and emotional profiles of individuals with PD-MCI. Additionally, the methodology of the usability study in PD-MCI patients undergoing cognitive training with PRoBio is presented.

The OPERA project focuses on cognitive interventions in PD-MCI, a condition that significantly affects attention, executive functions, memory, and visuospatial abilities (3, 5). To address the limitations of conventional rehabilitation, often lacking personalization, ecological validity and long-term adherence, the project adopts innovative, engaging, and technology-based solutions, as suggested in recent reviews (8, 9).

As a core component of this approach, the OPERA project first leverages the advantages of VR-based technology, which allows the creation of immersive or non-immersive, interactive, and customizable training environments (54, 55). These features foster patient engagement and provide high ecological validity (14, 56), which are key to promoting adherence (57) and enhancing neuroplasticity through multimodal stimulation and real-time feedback (21, 26, 58).

A further key component of the PRoBio platform is adaptive human-robot interaction, which is based on the integration of VR with physiological and behavioral measurements to personalize treatment. The integration of multimodal sensing, real-time adaptation, and socially interactive robotic feedback in PRoBio has the potential to offer a more personalized and responsive form of cognitive rehabilitation, tailored to the complex needs of individuals with PD-MCI. This unified, patient-centered system will henceforth offer a scalable, ecologically valid alternative to traditional rehabilitation, which will be particularly well-suited to early and sustained cognitive interventions in neurodegenerative disease.

Beyond its methodological and technological innovation, PRoBio carries relevant clinical and translational implications when compared with existing cognitive rehabilitation approaches adopting innovative technologies.

To the best of our knowledge, current robot-aided interventions in PD primarily deliver motor training with only secondary cognitive involvement (59, 60), whereas PRoBio explicitly targets cognitive–executive mechanisms through performance-adaptive, goal-oriented tasks. This represents a conceptual shift from movement-assisted to cognitive-based rehabilitation. Furthermore, PRoBio is designed to interpret biosignals as indicators of emotional and attentional states, thus allowing it to adjust not only task parameters but also the type and timing of feedback. PRoBio, hence, extends the paradigm of bio-cooperative interaction to encompass cognitive and affective domains. This feature has the potential to enhance engagement and adherence, two domains frequently impacted by apathy and variability in motivation in PD-MCI.

The rationale supporting PRoBio methodology could be found in recent clinical findings demonstrating that a personalized, multidisciplinary, and multimodal rehabilitation pathway integrating VR, robotics, and telerehabilitation produced significant improvements in cognitive and emotional functioning in patients with PD. This approach is not only effective but it is also highly usable, well-received by patients, and has the potential to enhance engagement and adherence. Adaptability through task adjustment, feedback, and progression monitoring, is proposed as a crucial determinant of usability and cognitive benefit (61). In line with the evolving literature, recent contributions highlighted the importance of evaluating not only motor and cognitive outcomes but also the psychosocial experience of technology use in neuromotor rehabilitation (62). PRoBio, hence, operationalizes principles of personalization and feedback adaptivity within a bio-cooperative closed-loop architecture to provide a data-driven, adaptive, and physiologically informed cognitive training.

Most importantly, the OPERA project proposes a cutting-edge approach that reflects the latest advancements in the field. According to human-centered design methodology, the development of the PRoBio platform has prioritized the understanding of the needs, preferences, and usability for the direct end users. Specifically, to ensure stakeholders' involvement, a PRoBio pre-development Focus Group and post-development Living Labs were conducted. An iterative process has been adopted: (i) the co-design of the platform based on structured focus groups with clinicians, caregivers, and patients; (ii) the technical integration of robotic, virtual, and physiological modules into a unified architecture; (iii) the living lab evaluation of system usability with healthy participants to refine interaction modalities; and (iv) the usability and exploratory efficacy assessment in individuals with PD-MCI.

The focus group has been conducted prior to any technical implementation (28). It has served as an initial step to explore the perspectives of PwPD, their caregivers, and healthcare professionals. Structured questionnaires have been used to investigate the perceived usability, value, and functional expectations of the future PRoBio platform. The purpose of this phase was to gather preliminary insights to inform the system's conceptual and functional design.

The Living Labs were intended to assess the usability of the PRoBio platform in a group of healthy participants, prior to assess its usability in PD-MCI patients. As the OPERA project focuses on a frail and potentially vulnerable population of patients, the Living Lab approach was appropriate for the preliminary testing and development of healthcare innovations in real-life care settings, as recommended by Zipfel et al. (63). Furthermore, the current literature supports this user-centered approach with aging populations to co-create effective and user-friendly health solutions (64). The active involvement of stakeholders (i.e., rehabilitation professionals and healthy volunteers) aimed to ensure that the platform was aligned with real-world needs and to promote its adoption in clinical practice. Based on the feedback gathered from Phases 1 and 3, a refined version of PRoBio has been developed. This staged approach ensured that potential technical issues could be identified and addressed without burdening vulnerable clinical populations. The last phase of the OPERA project (Phase 4. Usability study) specifically included individuals with PD-MCI, thus allowing the evaluation of usability in real-world rehabilitation contexts, and preliminary assessment of cognitive and functional outcomes.

Finally, the decision to alternate cognitive training protocols using an ABAB pattern was made to foster patients' compliance. The two protocols are balanced, allowing patients to train the same cognitive domains over the 4-week intervention without losing interest in the proposed tasks.

Some limitations should be acknowledged. The Living Labs involved a relatively small sample of healthy volunteers and healthcare professionals, which limited generalisability. However, this is consistent with the methodology of the Living Lab, where formal sample size calculation is not required (42). The absence of patients with PD-MCI may have reduced ecological validity, since healthy participants cannot fully reflect the challenges faced by individuals with motor and cognitive impairments. This choice was nonetheless intentional, as the Living Labs served as a preparatory step before the clinical testing. The subsequent phase of the OPERA project includes a clinical study on PD-MCI patients to directly evaluate usability, acceptability, and clinical applicability.

The Usability Study also presents some limitations. First, it involves a relatively small sample size of PD-MCI patients, limiting generalisability. However, this should be sufficient to conduct a preliminary usability analysis of the first refined PRoBio version. According to user-centered design, this version of the platform will undergo further refinement based on the results from the usability study in the clinical setting. In addition, patient-specific factors such as apathy, fatigue, and variability in compliance may influence engagement and adherence to the training. As a matter of fact, in PD-MCI patients, apathy has been shown to be associated with poorer cognitive performance (65). Future studies should take into account motivational and emotional profiles in PwPD to ensure reliable assessment of usability, safety, and clinical outcomes of a new technology (65, 66).

## 4 Conclusion

The OPERA framework suggests that a human-centered design approach is essential for developing, and continuously refining, technologies for cognitive rehabilitation. One of the main strengths of the project lies in its ability to propose an innovative, bio-cooperative solution. Bio-cooperative robotic systems constitute an emerging class of technologies enabling dynamic, user-centered interaction through multimodal interfaces (23). Their clinical feasibility has been previously demonstrated (22), highlighting the critical role of participatory design in fostering usability and acceptability. As reported by Yuan et al. (67), robot-assisted cognitive training offers a promising avenue for supporting individuals with cognitive impairments. However, challenges remain, including ethical concerns around safety and normative behavior, the need for stakeholder involvement at every stage of development, and the requirement for reliable, trustworthy, and cost-efficient systems.

The OPERA study contributes to addressing these challenges by integrating technological innovation with clinical research in cognitive rehabilitation for individuals with PD-MCI. Its user-centered approach, combined with a focus on usability, acceptability, and personalized digital feedback, provides valuable insights into the development of adaptive, scalable, and evidence-based digital health solutions tailored to this population.

From this perspective, the OPERA study aligns with the emerging paradigm of personalized rehabilitation, where treatment is dynamically adapted based on individual cognitive and psychophysiological profiles. Integrating robotics, VR, and biosensors into a cohesive rehabilitation protocol could potentially enhance outcomes not only for patients with PD-MCI, but also for individuals affected by other neurological conditions. Moreover, the project aligns with broader research efforts aimed at advancing bidirectional human–robot interaction as a foundation for future personalized rehabilitation protocols targeting cognitive-motor impairments in neurological conditions.

## Data Availability

The original contributions presented in the study are included in the article/Supplementary material, further inquiries can be directed to the corresponding author.
