# Supplementary material for: Integrated bio-cooperative robotic platform for virtual cognitive training in Parkinson's disease: design and methodology of the OPERA project
Source: Front Neurol. 2026 Jan 26;16:1680215. doi: 10.3389/fneur.2025.1680215 (PMC12883416; doi:10.3389/fneur.2025.1680215)
Supplement: Supplementary file 1 [file Data_Sheet_1.pdf]

Supplementary Material

1 Usability and Acceptability Questionnaires

A brief description of the 7 questionnaires to assess the usability and acceptability of PRoBio is provided below:

The System Usability Scale (SUS) is a 10-item self-report questionnaire designed to measure the perceived system usability. It evaluates key aspects of the user experience, such as:

- Ease of use, which explores the user's perception of how easy the system is to use in general;
- Familiarity and consistency, which assesses how intuitive or familiar the system is perceived;
- Overall satisfaction, which measures the level of acceptance and enjoyment derived from interacting with the system.

Participants respond to each item on a 5-point Likert scale ranging from “Strongly disagree” (1) to “Strongly agree” (5). The items alternate between positive and negative formulations to reduce the risk of automatic responses. Scores equal to or more than 85 indicate excellent usability, scores equal or under 50 indicate poor usability.

The eHealth Usability Benchmarking Instrument (HUBBI) is an 18-item self-assessment scale divided into 7 main factors that measure various aspects of usability in eHealth applications. These factors are:

- Basic System Performance (BSP), which assesses the speed, reliability, and stability of the system;
- Task-Technology Fit (TTF), which measures how well the system supports users in completing intended tasks;
- Interface Design (ID), which evaluates the visual aesthetics and usability of the interface;
- Navigation and Structure (NS), which examines how easy it is to navigate the system and understand its organization;
- Information and Terminology (IT), which analyzes the clarity, relevance, and comprehensibility of the information provided by the system;
- Guidance and Support (GS), which measures the accessibility and usefulness of the support resources available to users;
- Satisfaction (SAT), which assesses the overall level of satisfaction derived from using the system.

Participants respond to each item on a 5-point Likert scale, ranging from "Strongly disagree" (1) to "Strongly agree" (5). Normalized scoring goes from 0 (poor perceived usability) to 100 (great perceived usability).

The Short Version of the User Experience Questionnaire (UEQ-S) is an 8-item self-report questionnaire designed to quickly assess the user experience (UX) of a system. The items are divided into two main factors:

- Pragmatic Quality (PQ), which evaluates the system's effectiveness, efficiency and ease of use;
- Hedonic Quality (HQ), which measures pleasure and satisfaction derived from emotional engagement and aesthetics when using the system.

All items are presented as pairs of opposite adjectives (semantic differentials) and are rated on a 7-point Likert scale ranging from -3 to +3, with 0 indicating neutrality. Participants indicate their position between the two adjectives based on their personal experience with the system. Scores > +1.5 indicate a positive experience and scores < 0 indicate a negative experience.

The Scales for Perceived Usefulness and Perceived Ease of Use (TAM), are two 6-item self-report questionnaires designed to measure two key factors influencing technology acceptance:

- Perceived Usefulness, which assesses whether a user believes that using a specific technology will enhance their performance;
- Perceived Ease of Use, which evaluates whether the user believes that using the technology will be effortless.

Each statement is rated on a 7-point Likert scale ranging from "Strongly disagree" (1) to "Strongly agree" (7). High scores on Perceived Usefulness indicate that the user believes the technology will be beneficial for their work. High scores on Perceived Ease of Use suggest that the user perceives the technology as easy to learn and use.

The Italian Unified Theory of Acceptance and Use of Technology (I-UTAUT) is an adapted version of the original UTAUT model, developed to assess technology acceptance within Italian-speaking populations and cultural contexts. This self-report instrument evaluates key psychological constructs that influence individuals' behavioural intentions and their actual use of technology. The core components include:

- Performance Expectancy: the degree to which an individual believes that using the technology will enhance their job performance or goal achievement;
- Effort Expectancy: the perceived ease of use of the technology;
- Social Influence: the extent to which individuals perceive that important others believe they should use the technology;
- Facilitating Conditions: the perceived availability of resources and support to use the technology effectively.
- The I-UTAUT also includes measures of Behavioural Intention to use the technology and, in some versions, Use Behaviour. Responses are typically collected using Likert-type scales. The Italian version maintains the theoretical integrity of the original model while ensuring linguistic and cultural appropriateness through validated translation and adaptation procedures. It is commonly used in organizational, educational, and healthcare settings to assess users' acceptance of, and readiness for new digital tools and systems.

The total score is between 0 and 205, higher values indicate greater technology acceptance.

The NASA Task Load Index (NASA-TLX) is a self-report tool designed to measure an individual's perceived workload while performing a task. The questionnaire consists of six core dimensions, each assessing a specific aspect of workload:

- Mental Demand: the amount of mental and perceptual activity required by the task;
- Physical Demand: the physical effort needed to complete the task;
- Temporal Demand: the perceived time pressure during task execution;
- Performance: the individual's perception of their success in achieving task goals;
- Effort: the amount of exertion required to attain the desired performance level;
- Frustration: feelings of insecurity, irritation, and stress experienced during the task.

Each dimension is rated on a 20-point Likert scale ranging from "very low" to "very high". The total score is between 0 and 100, higher values indicate higher workload. Furthermore, the patient will indicate which of the dimensions has had more influence on the perceived workload. Using those values, it is possible to calculate a weighted total score.

The Technology Assisted Rehabilitation Patient Perception Questionnaire (TARPP-Q) is a self-report tool developed to evaluate patients' subjective experiences and perceptions associated with technology-assisted rehabilitation. It is specifically designed for clinical populations undergoing rehabilitative interventions that incorporate technological tools, such as virtual reality, robotics, or digital interfaces. The TARPP-Q explores multiple dimensions of patient perception, including:

- Emotional and motivational engagement, assessing how the technological component influences the patient's emotional response and motivation during rehabilitation;
- Perceived usability and accessibility, evaluating how intuitive, comfortable, and accessible the technology appears to the user;
- Therapeutic value, measuring the perceived effectiveness of the technology in supporting functional recovery and therapeutic goals;
- Psychological and physical impact, considering aspects such as fatigue, stress, or discomfort associated with the use of technology during therapy.

Responses are typically collected using a Likert scale. The TARPP-Q provides clinicians and researchers with valuable insights into patient-centered factors that may influence adherence, satisfaction, and the overall effectiveness of technology-enhanced rehabilitation programs. The Technology Assisted Rehabilitation Patient Perception Questionnaire (TARPP-Q) total scores range from a minimum of 46 to a maximum of 80, with subscale scores varying as follows: Positive Attitude (16–40), Usability (7–20), Hindrance Perception (5–12), and Distress (9–19).

2 Clinical, Cognitive and Psychological Assessment Measures

Below is a brief description of the measures used to evaluate patients' clinical characteristics, cognitive functions, and psychological state at T0 and T1 in the Pilot Study:

The MDS-Unified Parkinson's Disease Rating Scale (MDS-UPDRS). This is a standardized, validated clinical tool used to assess the severity and progression of Parkinson's disease (PD). It provides a more comprehensive and sensitive evaluation than the original UPDRS, covering both motor and non-motor symptoms.

The scale is divided into four parts:

Part I – Non-Motor Aspects of Daily Living

- IA: Clinician-rated non-motor symptoms (e.g., sleep, mood, cognition and autonomic functions)
- IB: Patient-reported non-motor symptoms

Part II – Motor Aspects of Daily Living: Patient-reported motor symptoms such as tremor, rigidity, and difficulties with walking or hand use

Part III – Motor Examination: Clinician-assessed motor function, including tremor, rigidity, bradykinesia, dyskinesia, and postural instability. This is evaluated in both "on" and "off" medication states, where applicable

Part IV – Motor Complications: Assesses therapy-related complications, including motor fluctuations and dyskinesias.

Each item is rated on a 5-point Likert scale (0 = no symptoms, 4 = severe symptoms), and the total score reflects the overall severity of the disease. Total scores range from a minimum of 0 to a maximum of 272, with the subscales scored as follows: Part I (Non-Motor Experiences of Daily Living) 0–52, Part II (Motor Experiences of Daily Living) 0–52, Part III (Motor Examination) 0–132, and Part IV (Motor Complications) 0–36.

The Parkinson's Disease Cognitive Rating Scale (PD-CRS). This is a neuropsychological tool designed to assess cognitive functioning in patients with Parkinson's disease (PD). The PD-CRS is clinically useful for tracking the progression of cognitive deficits in PD patients.

The scale includes 9 subtests targeting the following domains: executive functions, attention, verbal fluency, and visuo-constructional abilities. Specifically, it assesses:

- Immediate verbal memory recall
- Naming
- Sustained attention
- Working memory
- Clock drawing (free and copy)
- Delayed verbal memory recall
- Alternating verbal fluency

- Action verbal fluency

Each subtest has a specific scoring method and produces an equivalent score. The total score is calculated by summing the subtest results. These scores provide a comprehensive overview of the patient's cognitive status in PD. Total scores range from a minimum of 0 to a maximum of 134, assessing both subcortical and cortical cognitive functions, with higher scores indicating better cognitive performance.

The Stroop Color Word Test. This neuropsychological tool is used to assess selective attention, cognitive flexibility, and interference sensitivity, by measuring the ability to inhibit automatic responses. The test consists of three parts:

a) Word Reading – The subject reads color names (red, blue, green) printed in black ink.
b) Color Naming – The subject names the color of small circles (red, blue, green).
c) Interference Task – The subject is presented with color words printed in an incongruent ink color (e.g., the word "red" printed in blue ink) and must name the ink color, not the word.

The Stroop effect is assessed through two interference measures:

- Time interference effect: the difference between the time taken in the interference task and the average time taken in the first two tasks.
- Error interference effect: the difference in the number of errors made in the interference task compared to the average number of errors in the first two tasks.

This test provides insight into an individual's executive control processes, especially their ability to manage conflicting information. Higher scores in "Time Interference Effect" and in "Error Interference Effect" indicate worse attention and executive functions.

The Trail Making Test. This widely used neuropsychological test is valued for its simplicity and sensitivity to brain damage. It is composed of two parts:

Part A: The participant is asked to connect 25 numbered circles, scattered across a page in ascending numerical order (1–2–3…25).

Part B: The participant must alternate between numbers and letters in sequence (1–A–2–B…13–N), connecting 13 numbers and 12 letters randomly arranged on the page.

The tasks must be completed as quickly as possible, and the main outcome is the completion time for each part. If the participant makes an error, they are immediately asked to correct it without stopping the timer—thus, errors indirectly impact performance by increasing the total time. The Trail Making Test evaluates attention, cognitive flexibility, abstraction ability, psychomotor speed, executive functioning, and the capacity to adapt or shift an action plan. Scoring is based on the time (in seconds) taken to complete Part A and Part B, with no fixed maximum time but normative values varying by age group, with longer times indicating poorer performance.

The Parkinson's Disease Questionnaire (PDQ-39). It is a disease-specific instrument designed to assess quality of life in individuals with Parkinson's disease by evaluating the impact of the condition on daily living from physical, emotional, and social perspectives.

The questionnaire includes 39 items across 8 domains:

- Mobility (10 items): Assesses physical difficulties related to movement, such as walking and balance.
- Activities of Daily Living (6 items): Evaluates challenges in everyday tasks like dressing, cooking, and using one's hands.
- Emotional Wellbeing (6 items): Measures of emotional states such as anxiety, depression, and worry.
- Stigma (4 items): Captures feelings of social judgment and perceived stigma.
- Social Support (3 items): Assesses perceived support from family, friends, and caregivers.
- Cognitive Function (4 items): Investigates issues with memory, concentration, and mental clarity.
- Communication (3 items): Evaluates difficulties in expressing oneself and understanding others.
- Bodily Discomfort (3 items): Measures physical pain and discomfort.

For each item, the patient is asked to indicate how often a specific issue has affected them in the past 4 weeks, using a 5-point Likert scale.

Total score range is 0-100, with the following interpretation:

- 0-20: Great quality of life.
- 21-40: Fine quality of life, with some difficulties.
- 41-60: Life quality mildly compromised.
- 61-100: Life quality is compromised.

The 15-item Geriatric Depression Scale (GDS-15). It is a brief, validated screening tool specifically designed to assess depression in older adults. The GDS-15 comprises 15 yes/no questions selected for their ability to accurately detect depressive symptoms. It explores the affective, cognitive, and behavioral aspects of depression, such as loss of interest, feelings of worthlessness, fatigue, difficulty making decisions, and sadness.

Each response indicating a depressive symptom is scored as 1 point.

The total score range is 0–15, with the following interpretation:

- 0–4: No or minimal depressive symptoms,
- 5–8: Mild depression,
- 9–11: Moderate depression,
- 12–15: Severe depression.

The Geriatric Anxiety Inventory. It is a validated 20-item yes/no questionnaire designed to assess anxiety in older adults, including those with cognitive or physical impairments. It captures both somatic and psychological symptoms (e.g., worry, agitation and tension) while minimizing overlap with other conditions such as depression or neurological disorders. The questionnaire can be administered directly or with caregiver support.

Scores range from 0 to 20, with higher scores indicating greater anxiety severity.

Interpretation:

- 0–4: Minimal/No anxiety,
- 5–9: Mild to moderate anxiety,
- 10–14: Moderate anxiety,
- 15–20: Severe anxiety.

Table 1 – *Brief description of the two rehabilitation protocols (A and B). List of the selected specific tasks for each domain and, brief description of the interaction modality used – either touch screen or robotic arm*

**PROTOCOL A**	
**1. FIND THE DIFFERENCES**<br>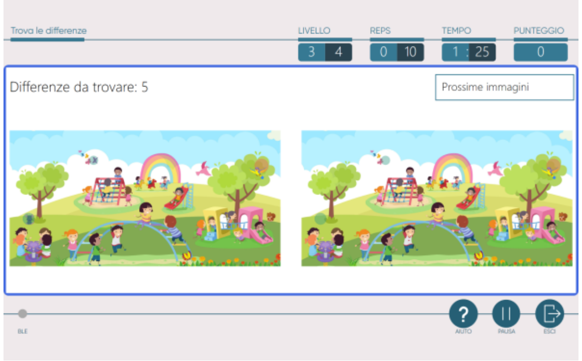 	**Cognitive Domain**: Attention<br><br>**Modality**: Touch screen<br><br>**Description**:<br><br>*Find the differences.* The patient, positioned in front of the screen, must look at the images and find the differences.<br><br>The score is assigned based on the number of errors made.<br><br>The exercise has 4 levels of difficulty and 3 presentation modes.
**2. CAR**<br>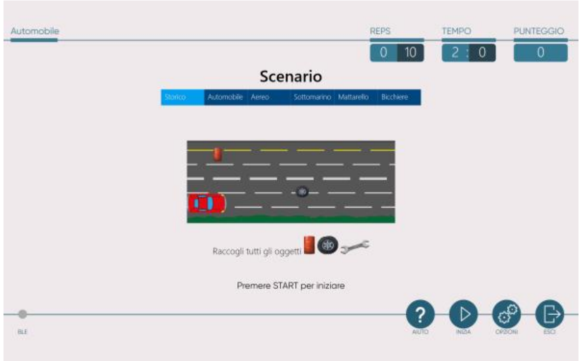 	**Cognitive Domain**: Attention<br><br>**Modality**: Robotic Arm<br><br>**Description**:<br><br>*Collect the indicated objects and avoid distractors.* The patient, positioned in front of the screen, must collect the objects by moving up and down.<br><br>The score is assigned based on the number of errors made.<br><br>The exercise includes 10 difficulty levels and 6 different scenarios.

**3. UMBRELLA**<br>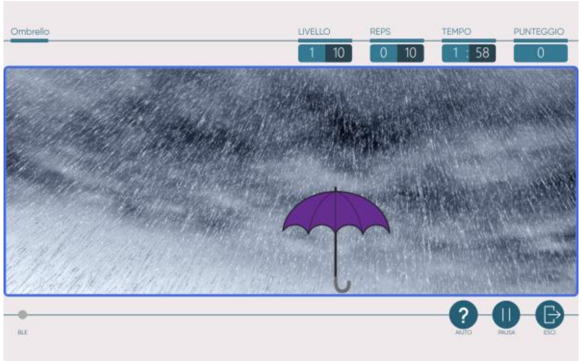	**Cognitive Domain**: Attention<br><br>**Modality**: Robotic Arm<br><br>**Description**:<br>*Collect the indicated objects and avoid the distractors.* The patient, positioned in front of the screen, must collect the objects by moving to the right and left.<br><br>The score is assigned based on the number of errors made.<br><br>No different difficulty levels are provided.
**4. CHANGING COLOR**<br>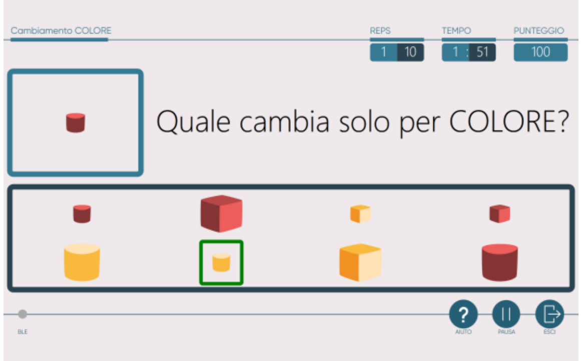	**Cognitive Domain**: Attention<br><br>**Modality**: Touch screen<br><br>**Description**:<br>*Find the element that differs only by color*. The patient, positioned in front of the screen, must select from the list at the bottom the element that differs only by color compared to the one shown at the top.<br><br>The score is assigned based on the number of errors made.<br><br>No different difficulty levels are provided, but 5 different modes are available.
**5. CHANGING SHAPE**<br>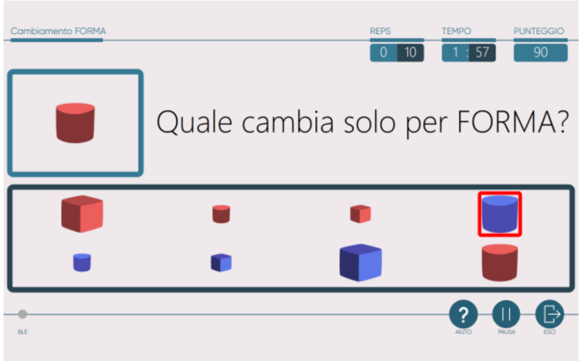	**Cognitive Domain**: Attention<br><br>**Modality**: Touch screen<br><br>**Description**:<br>*Find the element that differs only by shape.* The patient, positioned in front of the screen, must select from the list below the item that changes ONLY in shape compared to the item shown above.<br><br>The score is assigned based on the number of errors made.<br><br>There are no different levels of difficulty, but there are 5 different modes.

**6. REACTION TIME**<br>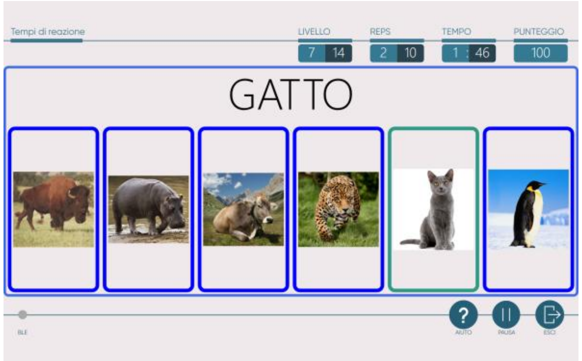	**Cognitive Domain**: Attention<br><br>**Modality**: Touch screen<br><br>**Description**:<br>*This exercise allows you to practise search and selection activities.* Reaction times are measured. The patient, positioned in front of the screen, must pay attention to the image or text presented and select the corresponding image as quickly as possible.<br><br>The score is assigned based on the number of errors made.<br><br>There are 14 levels of difficulty.
**7. SEQUENCES OF SHAPES**<br>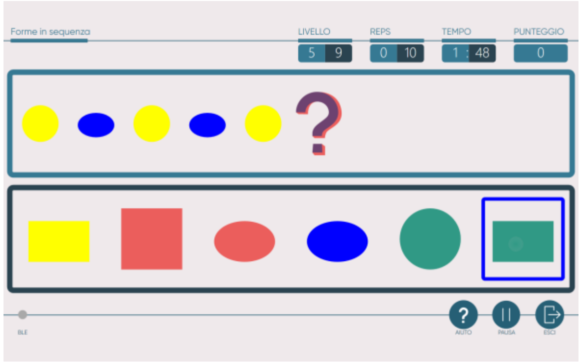	**Cognitive Domain**: Executive Functions<br><br>**Modality**: Touch screen<br><br>**Description**:<br>*Put the logical sequence together!* The patient, positioned in front of the screen, must put together the logical sequence shown at the top by selecting the correct objects from the list below.<br><br>The score is awarded based on the number of errors made.<br><br>There are 9 levels of difficulty.
**8. PLANNINGS**<br>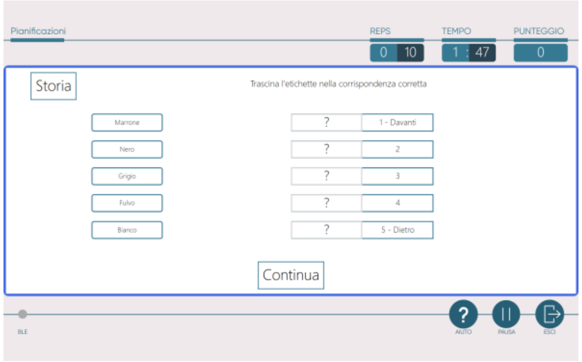	**Cognitive Domain**: Executive Functions<br><br>**Modality**: Touch screen<br><br>**Description**:<br>*Plan and organise tasks or objects according to the instructions provided.* The patient, positioned in front of the screen, must plan and organise tasks or objects according to the instructions provided.<br><br>The score is assigned based on the number of errors made.<br><br>There are no different levels of difficulty.

9. STREET PATH

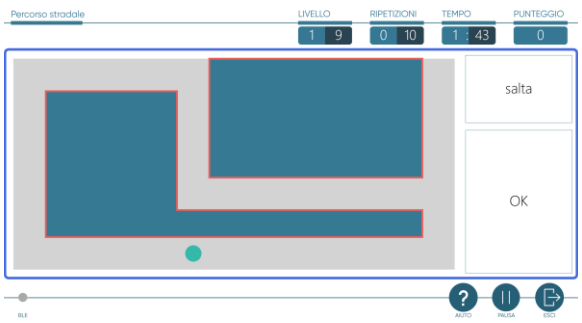

Cognitive Domain: Executive Functions

Modality: Touch screen

Description:
Memorise and repeat the route! The patient, positioned in front of the screen, must memorise the route shown on the map and try to repeat it.

The score is assigned based on the degree of similarity between the route presented and the one reconstructed.

The exercise has 9 levels of difficulty and 2 presentation modes.

10. COMPLETE THE SERIES

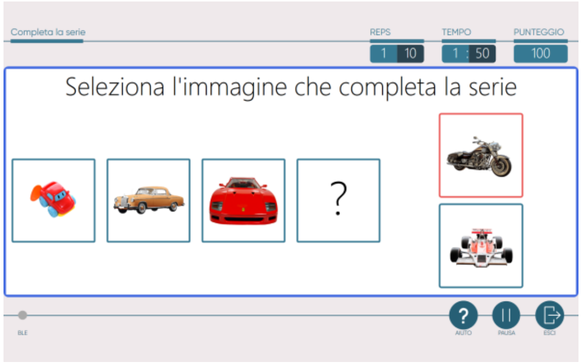

Cognitive Domain: Executive Functions

Modality: Touch screen

Description:
Select the image that completes the series. The patient, positioned in front of the screen, must select the image that completes the series.

The score is assigned based on the number of errors made.

There are no different levels of difficulty.

11. STROOP TEST

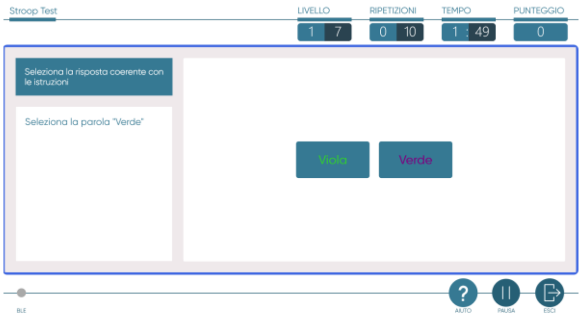

Cognitive Domain: Executive Functions

Modality: Touch screen

Description:
The patient, positioned in front of the screen, must indicate the word written in a specific colour of ink, regardless of its meaning or the word representing the name of the colour, ignoring the ink with which it is written.

The exercise has 7 levels of difficulty.

**12. FIND THE SYMMETRIC ITEM**<br>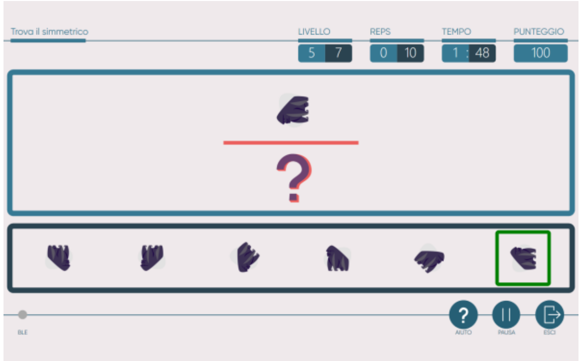 	**Cognitive domain**: visuo-spatial abilities<br><br>**Modality**: Touch screen<br><br>**Description**:<br>*Find the mirror image!* The patient, positioned in front of the screen, must select the symmetrical element from the list. The red line represents a mirror.<br><br>The score is assigned based on the number of errors made.<br><br>There are 7 different levels of difficulty.
**13. GUESS THE ROTATION DIRECTION**<br>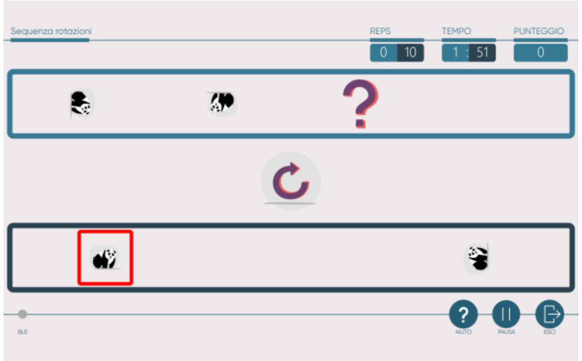 	**Cognitive domain**: visuo-spatial abilities<br><br>**Modality**: Touch screen<br><br>**Description**:<br>*Arrange the sequence in the indicated direction.* The patient, positioned in front of the screen, must select the objects in sequence to form a sequence of rotations in the indicated direction.<br><br>The score is assigned based on the number of errors made.<br><br>There are no different levels of difficulty.
**14. WORDS MEMORY**<br>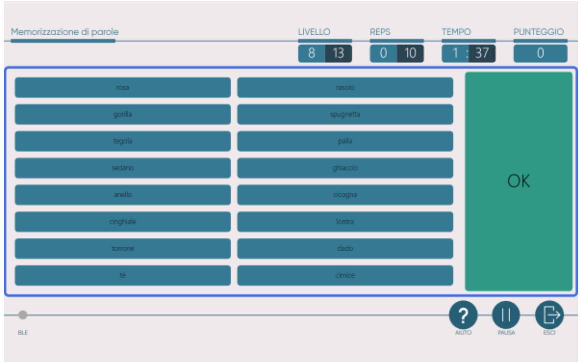 	**Cognitive domain**: Memory<br><br>**Modality**: Touch screen<br><br>**Description**:<br>*In this exercise, a set of words must be memorised.* The patient, positioned in front of the screen, must try to memorise the words that are presented. Subsequently, they select the words previously presented from the list provided.<br><br>The score is assigned based on the number of errors made.<br><br>There are 13 levels of difficulty.

PROTOCOL B

1. ATTENTIVE MATRIXES

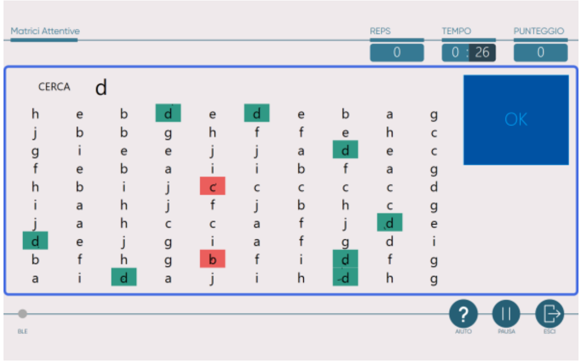

Cognitive Domain: Attention

Modality: Touch screen

Description:
Identification of elements proposed in matrices. The patient, positioned in front of the screen, must identify and mark the target elements proposed within a matrix of letters, numbers or symbols.

The score is assigned based on the number of errors made.

The exercise includes 12 levels of difficulty and 3 presentation modes.

2. UFO

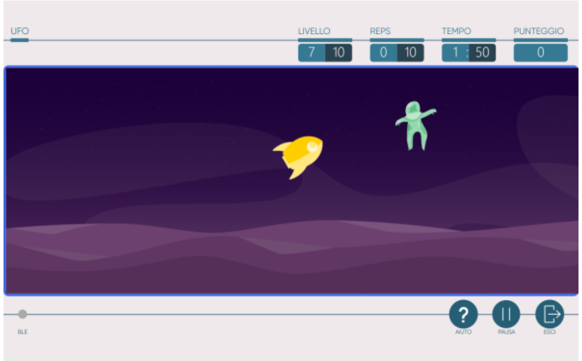

Cognitive Domain: Attention

Modality: Robotic Arm

Description:
Collect the indicated objects and avoid distractors. The patient, positioned in front of the screen, must collect the objects by moving in all directions.

The score is assigned based on the number of errors made.

There are no different levels of difficulty.

3. CONNECT THE POINTS– TMT

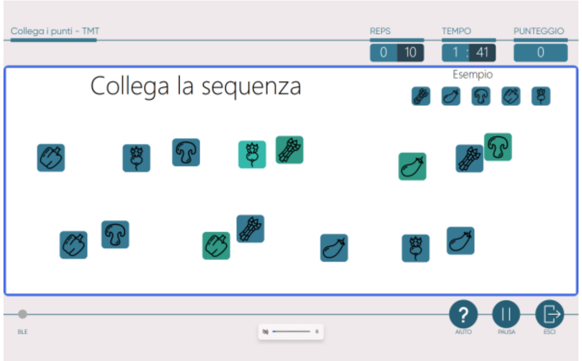

Cognitive Domain: Attention

Modality: Touch screen

Description:
Connect the dots according to the sequence presented. The patient, positioned in front of the screen, must connect the dots according to the sequence presented.

The score is assigned based on the number of errors made.

There is a single level of difficulty and four possible presentation modes.

4. CHANGING NOTHING

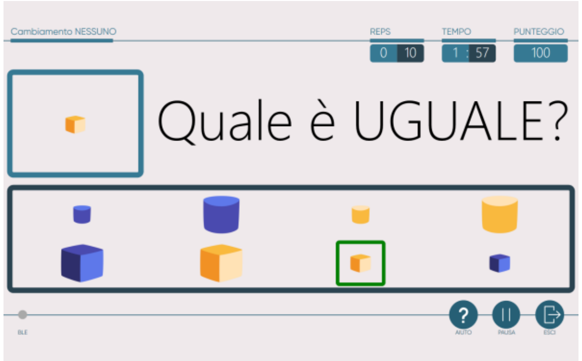

Cognitive Domain: Attention

Modality: Touch screen

Description:
Find the element that does not change. The patient, positioned in front of the screen, must select the element from the list below that does not change compared to the element shown above.

The score is assigned based on the number of errors made.

There are 5 different presentation modes.

5. CHANGING EVERYTHING

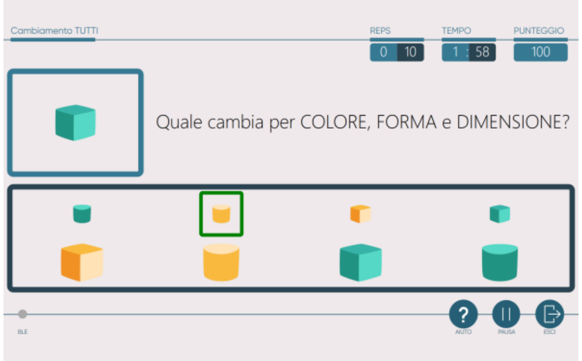

Cognitive Domain: Attention

Modality: Touch screen

Description:
Find the item that changes in shape, colour, and size. The patient, positioned in front of the screen, must select from the list below the item that changes in shape, colour, and size compared to the item shown above.

The score is assigned based on the number of errors made.

There are 5 different presentation modes.

6. REACTION TIMES

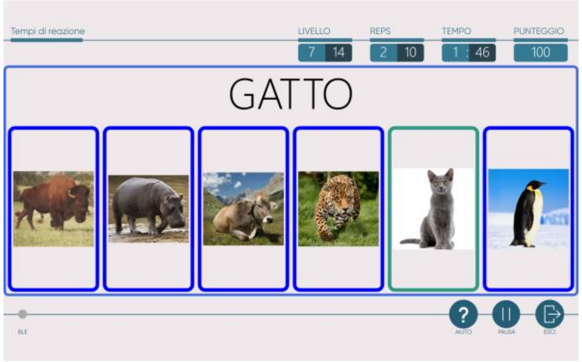

Cognitive Domain: Attention

Modality: Touch screen

Description:
This exercise allows you to practise search and selection activities. Reaction times are measured. The patient, positioned in front of the screen, must pay attention to the image or text presented and select the corresponding image as quickly as possible.

The score is assigned based on the number of errors made.

There are 14 levels of difficulty.

7. GET MONEY UP TO 10 EUROS

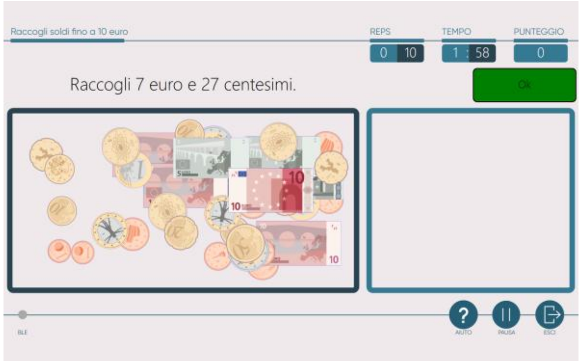

Cognitive Domain: Executive functions

Modality: Touch screen

Description:
Collect money until you reach the specified amount (1 euro, 10 euros, 100 euros and 1,000 euros). The patient, positioned in front of the screen, must drag the coins and banknotes shown on the left to the box on the right in order to collect the amount indicated at the top. When finished, they must press the OK button.

The score is awarded based on the number of errors made.

There are no different levels of difficulty for each amount.

8. SORT THE IMAGES IN SERIES

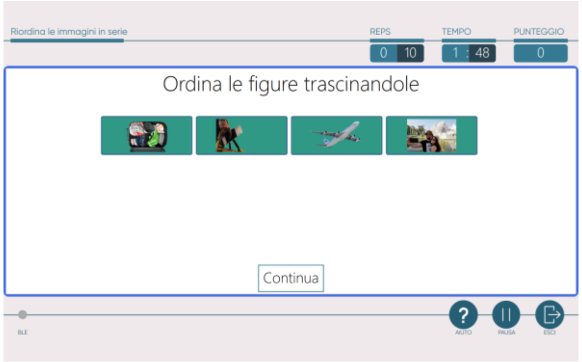

Cognitive Domain: Executive functions

Modality: Touch screen

Description:
Reorder the images by placing them in sequence. The patient, positioned in front of the screen, must reorder the images presented by placing them in sequence.

The score is assigned based on the number of errors made.

There are no different levels of difficulty.

9. DAILY SEQUENCES

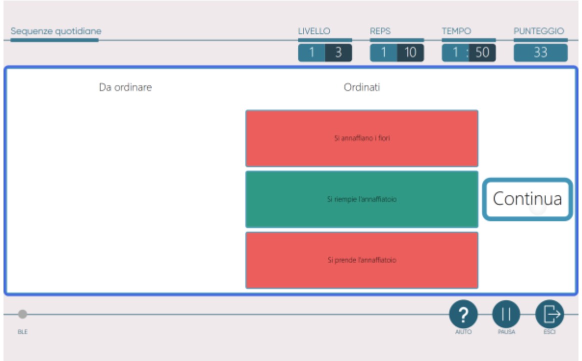

Cognitive Domain: Executive functions

Modality: Touch screen

Description:
Reordering daily actions. The patient, positioned in front of the screen, must put the daily actions presented in order.

Points are awarded based on the number of errors made.

There are three different levels of difficulty.

**10. DUAL CATEGORY**<br>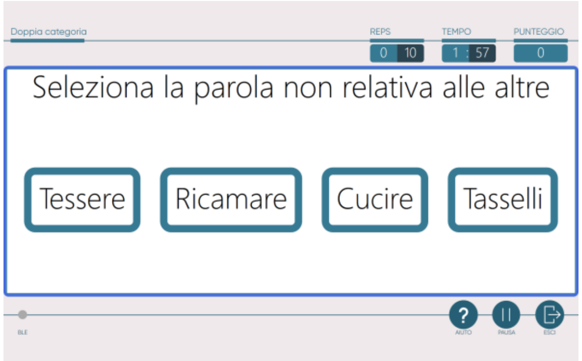 	**Cognitive Domain**: Executive functions<br><br>**Modality**: Touch screen<br><br>**Description**:<br>*Find the word that is not connected to all the others, paying attention to the double category.* The patient, positioned in front of the screen, must identify the word that is not connected to all the others presented.<br><br>The score is assigned based on the number of errors made.<br><br>There are no different levels of difficulty.
**11. ALPHANUMERIC ORDERING**<br>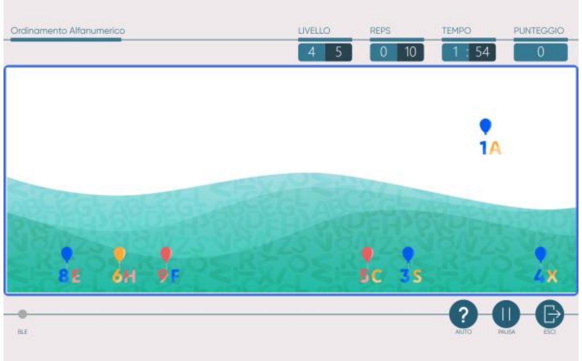 	**Cognitive Domain**: Executive functions<br><br>**Modality**: Robotic Arm<br><br>**Description**:<br>*Click on the balloons in ascending order based on their number or letter.* The patient, positioned in front of the screen, must click on the balloons presented, in ascending order based on their number or letter.<br><br>The score is assigned based on the number of errors made.<br><br>There are 5 levels of difficulty and 2 presentation modes.
**12. RECOGNIZE THE OBJECT AMONG MANY**<br>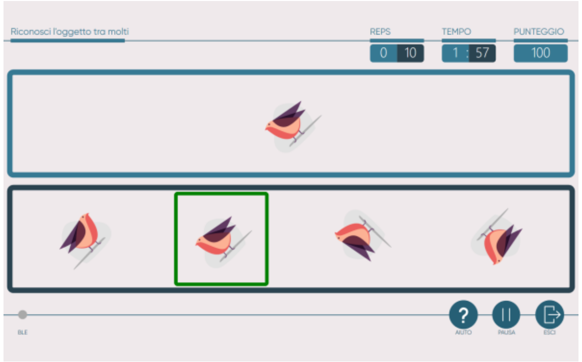 	**Cognitive Domain**: Visuo-spatial abilities<br><br>**Modality**: Touch screen<br><br>**Description**:<br>*Recognise the object among a set of similar objects that have been rotated and/or mirrored.* The patient, positioned in front of the screen, must select the item from the list that is identical to the one indicated.<br><br>The score is assigned based on the number of errors made.<br><br>There are 7 levels of difficulty.

13. PUZZLE

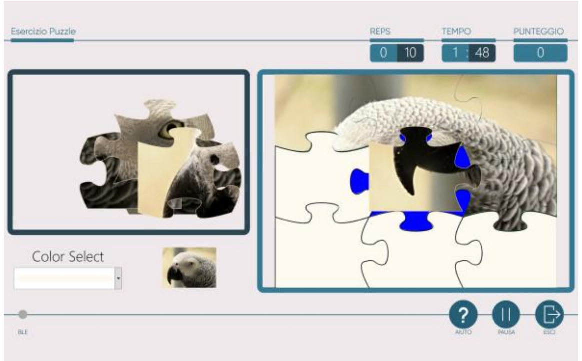

Cognitive Domain: Visuo-spatial abilities

Modality: Touch screen

Description:
Complete the puzzle. The patient, positioned in front of the screen, must drag the pieces of the puzzle and compose the image they see at the bottom left.

The score is assigned based on the number of errors made.

There are 10 presentation modes.

14. LONG TERM MEMORY

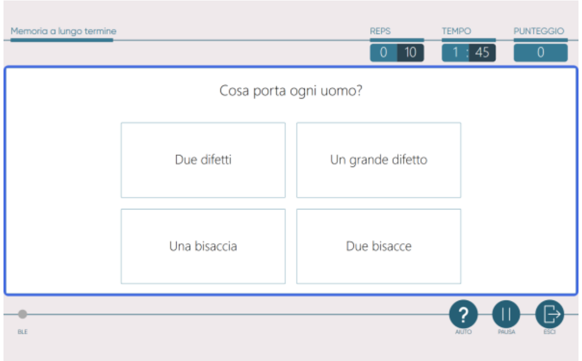

Cognitive Domain: Memory

Modality: Touch screen

Description:
Reading a passage with questions. The patient, positioned in front of the screen, must carefully read the passage presented and answer the related questions.

The score is assigned based on the number of errors made.

There are 4 levels of difficulty.
